# Antibacterial property of hydroxyapatite extracted from biological sources and doped with Cu^2+^ and Ag^+^ by Sol-gels method

**DOI:** 10.1038/s41598-025-89886-1

**Published:** 2025-04-09

**Authors:** Mahsa Abbasi, Mehdi Rashnavadi, Milad Gholami, Somayeh Molaei

**Affiliations:** 1 Department of Chemistry, Faculty of Basic Sciences, Ilam University, Ilam, Iran; 2 Faculty of Veterinary Medicine, Ilam University, Ilam, Iran; 3 Biotechnology and Medicinal Plants Research Center, Ilam University of Medical Sciences, Ilam, Iran; 4 Pharmaceutical Sciences Research Center, Kermanshah University of Medical Sciences, Kermanshah, Iran; 5 Department of Chemistry, Faculty of Science, University of Kurdistan, Sanandaj, Iran

**Keywords:** HA extraction, Thermal decomposition, HA doped with copper and silver, Sol-gel method, Antibacterial properties, Biochemistry, Biosynthesis

## Abstract

Hydroxyapatite is used in dental materials such as fillings, veneers, and implants due to its biocompatibility and similarity to the natural tooth structure. Goat teeth contain a high amount of hydroxyapatite, the primary mineral component of tooth enamel. Hydroxyapatite in goat tooth enamel ranges from 70 to 90%, sheep teeth from 75 to 85%, and cow teeth from 60 to 75% by weight. Sheep and Cow teeth have slightly lower hydroxyapatite content compared to goats, which is due to slight differences in their species. Hydroxyapatite can be modified or combined with other compounds (metals) to become a compound with high antibacterial properties, which have various applications in dentistry and medicine. This study aims to extract HA particles from the teeth of domestic animals (goats, sheep, and cows), especially goats (higher hydroxyapatite) in a shorter period, purer and with a simpler and more economical method and without adding chemical compounds containing Phosphate was compared to other methods and HA extracted from goat teeth ash was doped with copper and silver metal ions (HA-Doped Cu^2+^-Ag^+^) and showed high antibacterial properties. The ideal temperature for calcining HA is 1150 °C for 2 h. The physical properties of extracted HA and HA-Doped Cu^2+^-Ag^+^ were investigated using X-ray diffraction and Fourier transform infrared spectroscopy (FT-IR). It was observed in SEM that the distribution of diameter and size is different depending on the molar ratio of Cu^2+^ and Ag^+^. According to the XRD report, copper metals entered the network and silver metal was placed around the hydroxyapatite. Antibacterial activity was effective using agar and tube diffusion methods. Minimum inhibitory concentration (MIC) and minimum antibacterial concentration (MBC) were determined. HA doped with copper and silver is an effective antibacterial agent. It proves to be the most effective antibacterial agent in biomedical applications and is compatible with all bacterial strains.

## Introduction

Hydroxyapatite with the formula (Ca_10_ (PO_4_)_6_(OH) _2_) is a biocompatible and bone conductive material for orthopedic surgery and bone grafting. Which has wide applications in the field of biological materials, dentistry and medicine. It can fill bone defects, improve integration between implants, promote the growth of new bone tissue, and help repair and restore damaged and lost bone. Hydroxyapatite-based bone substitutes and scaffolds provide a suitable environment for the attachment and differentiation of bone cells, thereby promoting bone healing and regeneration. Its other characteristics include thermal stability, excellent bone conductivity, biocompatibility and non-toxicity^1,2^. HA can be obtained synthetically or naturally from chemical, biological sources, or waste. Natural extraction of HA from biological sources, such as teeth and bones^3^. Marine sources, such as fish bones and fish scales, shell sources like sea shells^4^ eggshells^5^ plants^6^ algae, and mineral resources such as limestone^7^.

Effective removal of biological waste from the environment is essential to the preservation of public health, the protection of natural ecosystems, and sustainable development. The appropriate method and technology must be chosen by the nature and scale of biological waste, as well as specific environmental and regulatory considerations in the region^8^.

HA can be successfully processed using various chemical methods, such as emulsions^8^, sol-gels^9^, thermal synthesis^10^, wet chemical precipitation^11^, microwaves^12^, and hydrothermal processes^13^.

Thermal processes usually involve high temperature calcination and produce highly crystalline and pure hydroxyapatite. Parameters such as temperature, heating rate and calcination time can be adjusted to produce desired shapes (spheres, rods, plates) and different sizes. Other synthesis techniques often causeless control of the final form of hydroxyapatite. Thermal methods are generally easier to mass produce than other synthesis techniques^14,15^.

Thermal methods are distinguished by their ability to produce high-crystalline, pure, and morphologically controlled hydroxyapatite with excellent mechanical properties. Furthermore, thermal technology offers advantages in terms of scale, reproducibility, and versatility, making it a preferred choice for the synthesis of materials based on hydroxyapatite for various biomedical and dental applications^16,17^.

In these processes, the sol-gel route is considered a desirable method of preparation compared to other processes because of its low crystallization temperatures, which reduce defects, and improve structural integrity^14,16^. Furthermore, sol-gel methods are one of the most common synthesis techniques due to their flexibility, the ability to replace ions in materials, excellent chemical uniformity, low cost, and ease of operation^17–19^.

In addition, metal doping on HA particles, such as copper and silver, can often be achieved through sol-gel technology, resulting in strong antibacterial properties^20,21^.

Sol-gel technology is considered a simple and effective method for adjusting nanomaterial structures and properties. It is more suitable for mixing heat-sensitive impurities. It produces hydroxyapatite with higher surface area and porosity, which may be useful for specific biomedical applications^21–24^.

Some commercial applications of HA include its use in dental, maxillofacial, and orthopedic surgery, drug delivery systems, metal prosthetic coatings, bone cement, and fillers. However, powder HA cannot be used for load support applications due to its low mechanical stability and brittleness^25^.

Furthermore, bacterial infections of HA ceramics during or after implantation have become a significant issue that can lead to prolonged pain and implant failure^26^. HA material is mainly composed of calcium and phosphate and is not resistant to bacterial infections.

For this reason, the preparation of hydroxyapatite with antibacterial properties has been considered^27^.

Atomic replacement or doping of HA with specific ions (metal) is an attractive approach to increase its antibacterial properties and increase the mechanical strength of bio ceramic materials^28^.

In many studies, substitution of metals in the hydroxyapatite network has been reported^29,30^. Several studies on HA modified by Ag^+^ metal have shown broad-spectrum antibacterial efficacy^31–34^. However, high concentration of Ag^+^ in HA causes allergy and increased cytotoxicity^35^. The use of metal ions such as Cu^2+^ helps to reduce these negative effects and maintain the optimal antibacterial effect of Ag^+^. Cu^2+^ strengthens the metabolism by facilitating the production of interconnected collagen and increasing enzyme activity^36^.

In addition, the use of Cu^2+^ can produce toxic biomaterials with excellent antimicrobial properties and mechanical integrity^37–40^.

In this research, the aim of extracting hydroxyapatite from the teeth of domestic animals (goats, sheep and cows), especially goats, by thermal decomposition at 1150 °C, and then sol-gel doping of metals (copper and silver) on hydroxyapatite from goat teeth, which No such report has been given so far. Antibacterial properties of hydroxyapatite doped with metals (copper and silver) against gram positive and negative bacteria are investigated.

## Experimental details

### Extraction of HA from goat, sheep and cow teeth by thermal decomposition method

The thermal decomposition method can be used to extract hydroxyapatite from goat, sheep, and cow teeth and bones. This method involves exposing the samples to high temperatures to decompose the organic components and separate the inorganic hydroxyapatite.

The jaw bones of domestic animals (goat, sheep, and cow) were collected from authorized slaughterhouses that were slaughtered. The teeth were then separated from the jawbone. To prevent the formation of soot and remove organic substances and collagen, the teeth were boiled in distilled water, and alcohol and acetone were used for degreasing. The impurities adhered to the teeth were scraped and cleaned using a dental spatula. Boiled in distilled water for 30 min. The obtained samples are clean and free of any soft tissue or contaminants. In the next step, the teeth were dried for one day either in the sun or in a laboratory dryer (oven) at 25 °C for 24 h to evaporate the absorbed moisture.

After drying the samples, grind them using a ball mill until they form a fine powder. This increases the surface area and facilitates the thermal decomposition process. Place the teeth in the crucible inside the thermal furnace at 650 °C, 850 °C, and 1150 °C for 2 h. At 351 °C, a significant amount of vapor was emitted from the sample, suggesting gas release from the tooth. We conducted this cooking process to ensure the thorough removal of organic matter, guarantee the safety of the material, and prevent microbial contamination. When the samples were removed from the furnace. Black ash was observed at 650 °C, indicating the presence of carbon and impurities. For this reason, the baking time was increased, and the samples were heated for 2 h at 900 °C and 1150 °C. Until they are completely calcined.

During baking, the color of hydroxyapatite changed from black and gray to white. Natural hydroxyapatite is the name of this material. In the field of biology, hydroxyapatite carbonate is considered a widely used and essential component. This is a key advantage of preparing carbonated hydroxyapatite. Grind the collected residue into a fine powder once more. This step helps achieve a uniform particle size and removes any larger debris. In addition, if desired, the powder can be sieved to obtain a more homogeneous particle size distribution. Wash the powder with distilled water to remove any remaining impurities or contamination. Repeat the washing process several times to ensure thorough cleaning. After washing, allow the powder to air dry or use a low-temperature oven to remove moisture. Store HA in a dry, airtight container to prevent moisture absorption.

The thermal decomposition method can alter the crystal structure and properties of extracted hydroxyapatite. Therefore, accurate temperature control and optimization of process parameters are necessary to obtain high-quality hydroxyapatite.

### Copper (Cu2+) and silver (Ag+) nitrate doping on HA

HA can be doped or modified with various elements to create specific properties or enhance its performance. Doping HA with Ag^+^ and Cu^2+^ is one of the approaches used to enhance antimicrobial properties.

First, hydroxyapatite was extracted from teeth (goat (HA-TG), sheep (HA-TSh) and cow (HA-TC)) using thermal method (1150 ℃). Doping process on hydroxyapatite extracted from goat (HA-TG) teeth with copper and silver was done by sol-gel method. In this way, 50 mg of hydroxyapatite extracted from goat teeth was weighed, then dissolved in water and 70% ethanol and placed on a magnetic stirrer at 50 °C. Silver nitrate and copper nitrate were added in the same ratio (25 mg) to the reaction mixture, and the medium was acidified with 1 N HCl. Leave the mixture overnight for the reaction to complete. Then the sample was centrifuged and washed three times with water and ethanol in equal proportions. Washing was done to remove free metals (copper and silver) that did not enter the hydroxyapatite network. Then put it in the oven at 50 ℃ until it dries completely. Hydroxyapatite doped with copper and silver was obtained.

### Morphological study

#### Scanning electron microscope (SEM)

SEM, magnifying more than 300,000x and even 1,000,000x, analyzes inorganic and organic materials at the nanometer (nm) to micrometer (µm) scale. Different points on the surface were selected for photographing using a scanning electron microscope (SEM) (JSM-840 A, JEOL, Japan). In fact, in this method, the image is captured by electrons, which have a much higher magnification power than optical microscopes and can be used for thousands of imaging cycles. Porosity and surface roughness were measured^32^. In a Field Emission Scanning Electron Microscope (FE-SEM), more detailed images can be observed with a resolution of 1 to 0.5 nanometers, as compared to a standard SEM^41^.

### Chemical, Physical, and mechanical characterizations

#### Fourier Transform Infrared Spectrometer (FTIR)

Nicolet (Bruker Germany Vertex 70 FT-IR) spectrum (FTIR) was used to identify functional groups in compounds. Its wave number ranges from 100 to 4000 cm^− 1^.

#### Inductively coupled plasma mass spectrometry (ICP)

The chemical compounds in the samples were analyzed using inductively coupled plasma optical emission spectrometry (ICP-MS) Agilent 7500 series^42^.

#### XRD analysis

Detailed information about a material’s physical properties, crystallographic structure, and chemical composition was obtained using X-ray diffraction (XRD), a non-destructive technique^43^.

#### Thermal gravimetric analysis (TGA)

To investigate the mass changes of the samples due to temperature increase, they were measured and analyzed using a TGA-DTG device manufactured by TA Instruments in America.

TGA was performed in an argon atmosphere with a net flow rate of 100 mL/min. A thermocouple was placed inside the reactor to monitor the wall temperature. To obtain TGA data, 6 mg of each sample was loaded into the TGA heating zone. The sample was heated to 105–110 °C for 10 min and maintained at 110 °C for an additional 30 min to determine the percentage of weight loss related to moisture^44^.

### Antibacterial test on hydroxyapatite doped with copper (Cu2+) and silver (Ag+)

Antibacterial tests were conducted on strains of Staphylococcus aureus (ATCC 13813) and Bacillus cereus (ATCC 33591) from gram-positive bacteria, as well as on Escherichia coli (ATCC 25922) and Pseudomonas aeruginosa (ATCC 27853) from gram-negative bacteria. The microbial strains mentioned were obtained from the Regional Center of Collection of Industrial Fungi and Bacteria of Iran in Tehran, Iran.

Agar diffusion and disc diffusion methods were used to assess the antimicrobial activity. For this purpose, a 0.5 McFarland agar suspension was prepared from bacteria cultured for 24 h on Mueller Hinton culture medium in Mueller Hinton broth culture medium. Then, 1 mL of the suspension of each bacterium was cultured using the pure plate method. After cultivating the desired bacteria in the form of grass on the surface of the plate containing Mueller Hinton agar culture medium, sterile discs prepared with sterile forceps are placed on the surface of the plate infected with bacteria. After complete contact with the culture medium at a suitable distance from each other, powder hydroxyapatite doped with Cu^2+^ and Ag^+^ as well as diluted powder with varying concentrations of hydroxyapatite doped with Cu^2+^ and Ag^+^, are poured onto the discs. Dilution was done with 10% dimethyl sulfoxide (DMSO). After 24 h of incubation at 37 °C, the diameter of the growth inhibitory halo was measured. Also, the antimicrobial effect of this substance was investigated in comparison with tetracycline and gentamicin antibiotic discs. The test to determine the antimicrobial effect was performed with three repetitions, and the average antimicrobial activity was reported^45^.

### Determining the minimum concentration of inhibition (MIC) and the minimum concentration of bactericide (MBC)

The tube dilution method was used to determine MIC and the minimum lethal concentration. To determine the MIC of the suspension of bacterial strains prepared from the 12 h culture medium, they were compared with a McFarland standard turbidity of 0.5. The initial concentration was 50 µL, and successive dilutions up to 5 dilutions were prepared and added to the microwells containing the liquid culture medium. Some microwells containing only microbial suspension without hydroxyapatite doped with Cu^2+^ and Ag^+^ were considered as controls. Then the 96-well microplate was shaken for 20 min and placed in an incubator at 35–37 °C for 24 h. After this incubation period, the mentioned time, MIC was determined by observing the turbidity in the microwells. This was done by comparing the turbidity changes of the liquid culture medium with those of the control. The first, the first well in which no microbial growth was observed was considered the MIC. Then, 20 µl of each MIC dilution and several higher dilutions were transferred to the Mueller Hinton Agar culture medium. Each plate was specific to a particular bacterial species. The plates were then placed in an incubator for 24 h. After this period, the first dilution without any bacterial growth was identified to determine the MBC.

Hydroxyapatite powder doped with Cu^2+^ and Ag^+^ in various concentrations (50, 100, 200, 400 and 800 mg/ml) was found to be effective against Escherichia coli, Pseudomonas aeruginosa, Staphylococcus aureus, and Bacillus cereus using the agar diffusion method and tube dilution. Antibiotics tetracycline and gentamicin were used as positive controls, while DMSO served as the negative control. The MIC and MBC were determined^46^.

## Results

### Morphological study

#### Scanning electron microscopy SEM, FE-SEM, and Histogram analysis

**Fig. 1:**
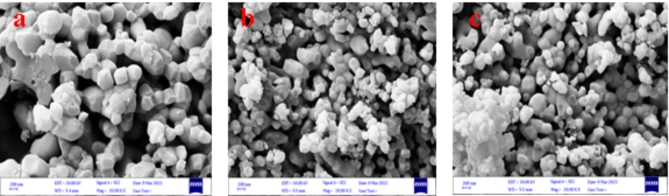
SEM- Hydroxyapatite (**a**) Goat teeth at 1150 °C (**b**) Sheep teeth at 1150 °C (**c**) cow teeth at 1150 ℃.

Figure 1 shows the SEM images of HA obtained from the ash of goats, sheep, and cow teeth at 1150 °C. Crystal sizes ranging from 171.2 to 485.4, and 515.8 nm were observed. In sheep teeth ash, crystal sizes varied from 60.29 to 267.1 nm, with porosity levels ranging from 1.446 to 1.670 and 2.349 μm as shown in Fig. 1 at 10 KV. Morphology shows that the size of crystals increases with increasing temperature^47^.

**Fig. 2:**
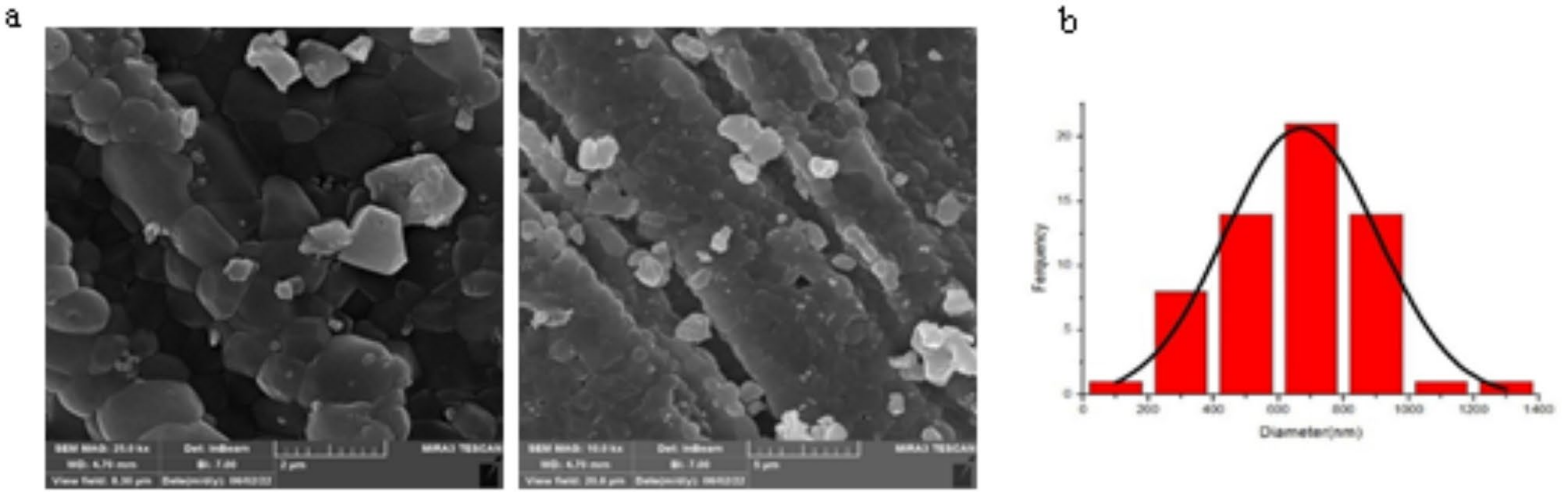
(**a**) FE-SEM - hydroxyapatite of Goat teeth at 1150 ℃. (**b**) An average particle size histogram for Goat teeth at 1150 ℃.

Figure 2 shows the FE-SEM diagram of hydroxyapatite extracted at 1150 °C from goat teeth. Its hexagonal shape is evident, with lengths of 1319.057 nm and 50.873 nm. Histogram analysis reveals the average particle size obtained from FE-SEM^48^.

**Fig. 3:**
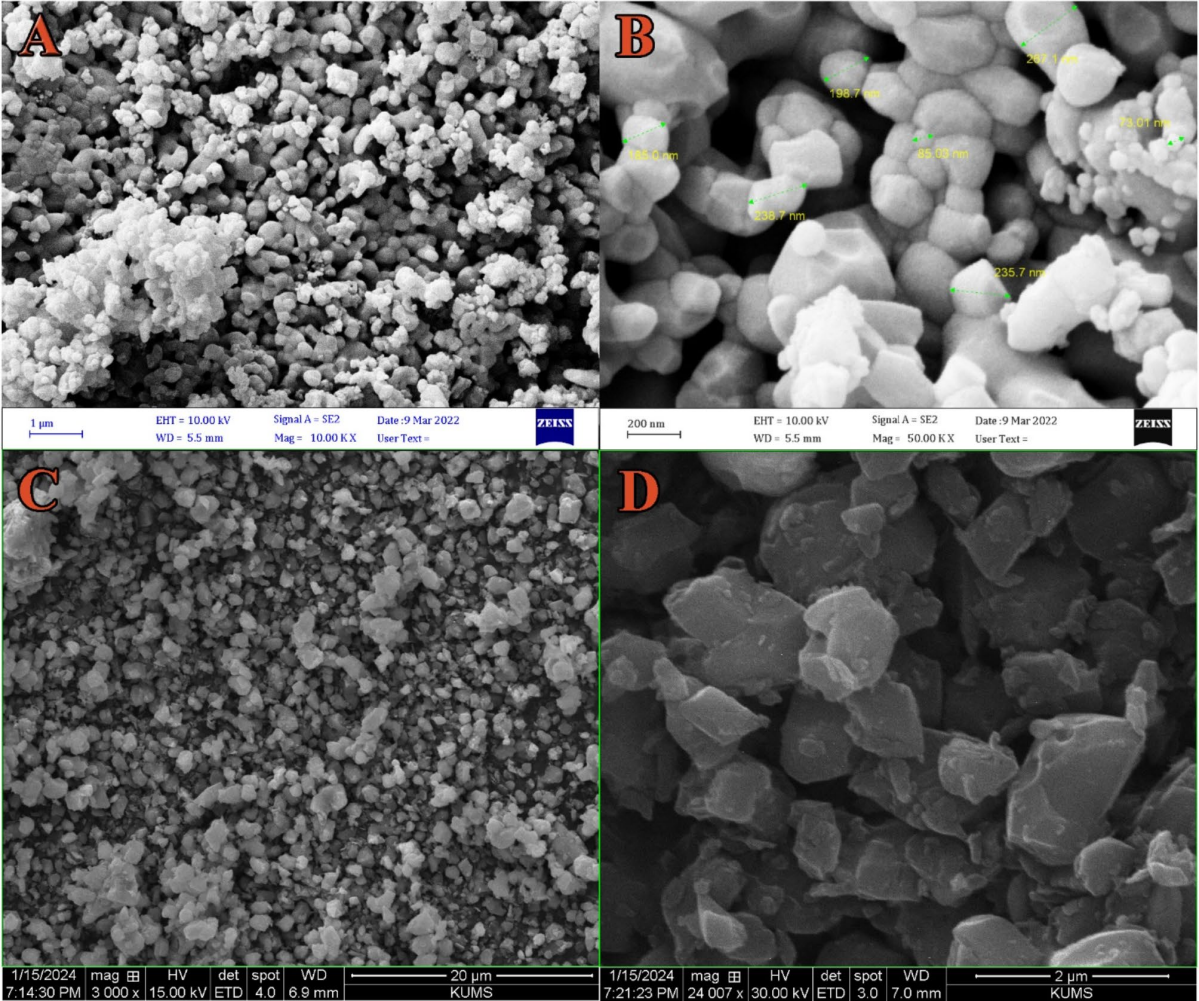
SEM of hydroxyapatite (Goet teeth) doped with Cu^2+^ and Ag^+^.

Figure 3 shows that the morphology and microstructure of hydroxyapatite (HA) extracted from goat teeth and doped with Ag^+^ and Cu^2+^ metal ions were evaluated using scanning electron microscopy (SEM). The surface morphology of undoped hydroxyapatite (HA) in images A and B shows numerous agglomerates, which give rise to small spherical particles with an average diameter ranging from 73.01 to 267.1 nm. Images C and D indicate that the presence of Cu^2+^ and Ag^+^ did not significantly affect the morphology of HA after doping. These images show that all the samples consist of similar agglomerates and small crystals. Moreover, the micrograph provides insight into the interconnected micro-particle agglomerates, which exhibit various shapes, morphologies, and distributions. However, the uniformity, size, and distribution of these nanoparticles vary depending on the nature of the doping metal ion.

### Chemical, Physical, and mechanical characterizations

#### Infrared spectroscopy (FTIR)

**Fig. 4:**
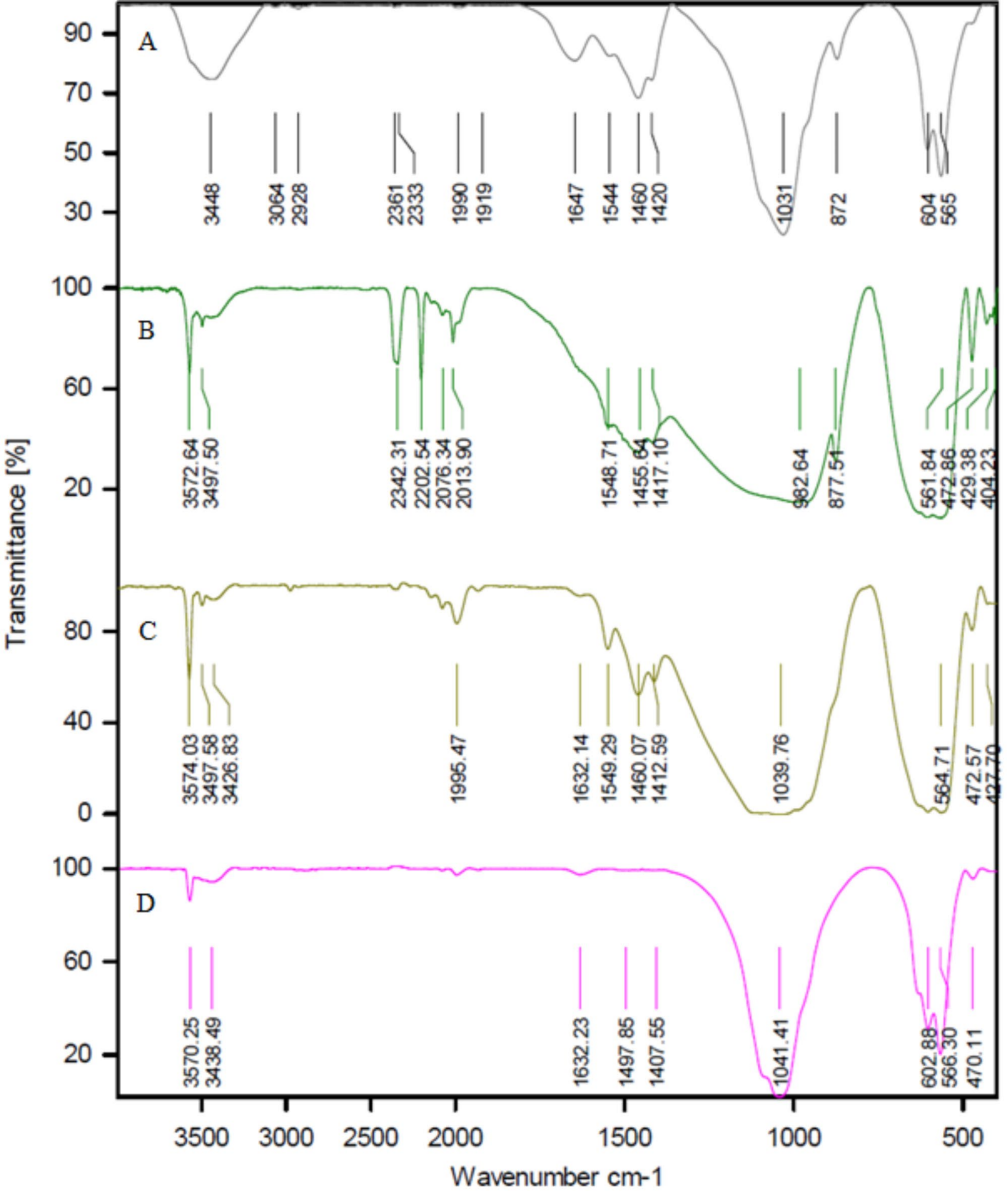
FTIR - Goat teeth ash (HA-TG) at A: 0 ℃ B: 600 ℃ C: 900 ℃ D: 1150 ℃.

**Fig. 5:**
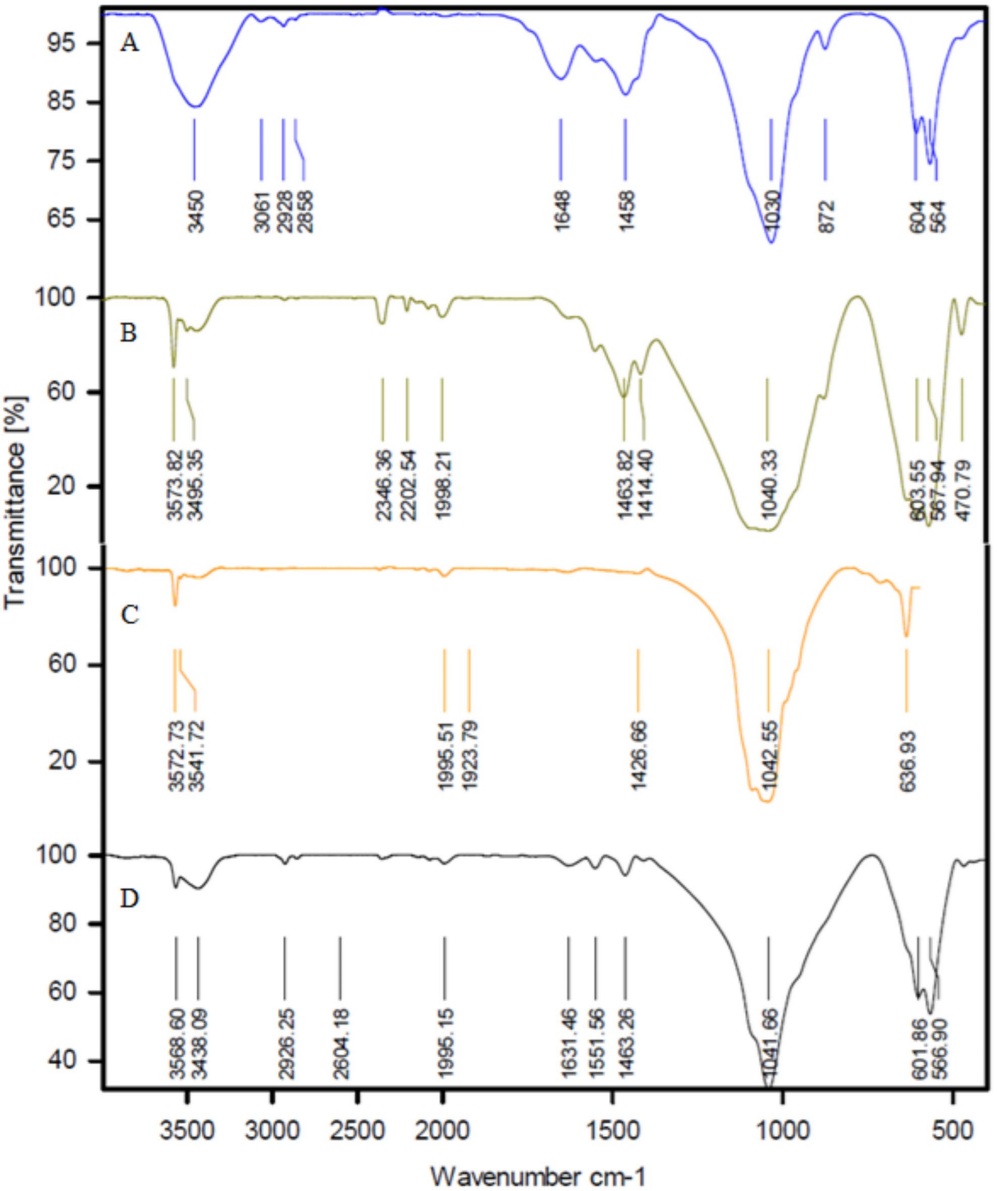
FTIR – Cow teeth ash (HA-TC) A: 0 ℃ B: 600 ℃ C: 900 ℃ D: 1150 ℃.

**Fig. 6:**
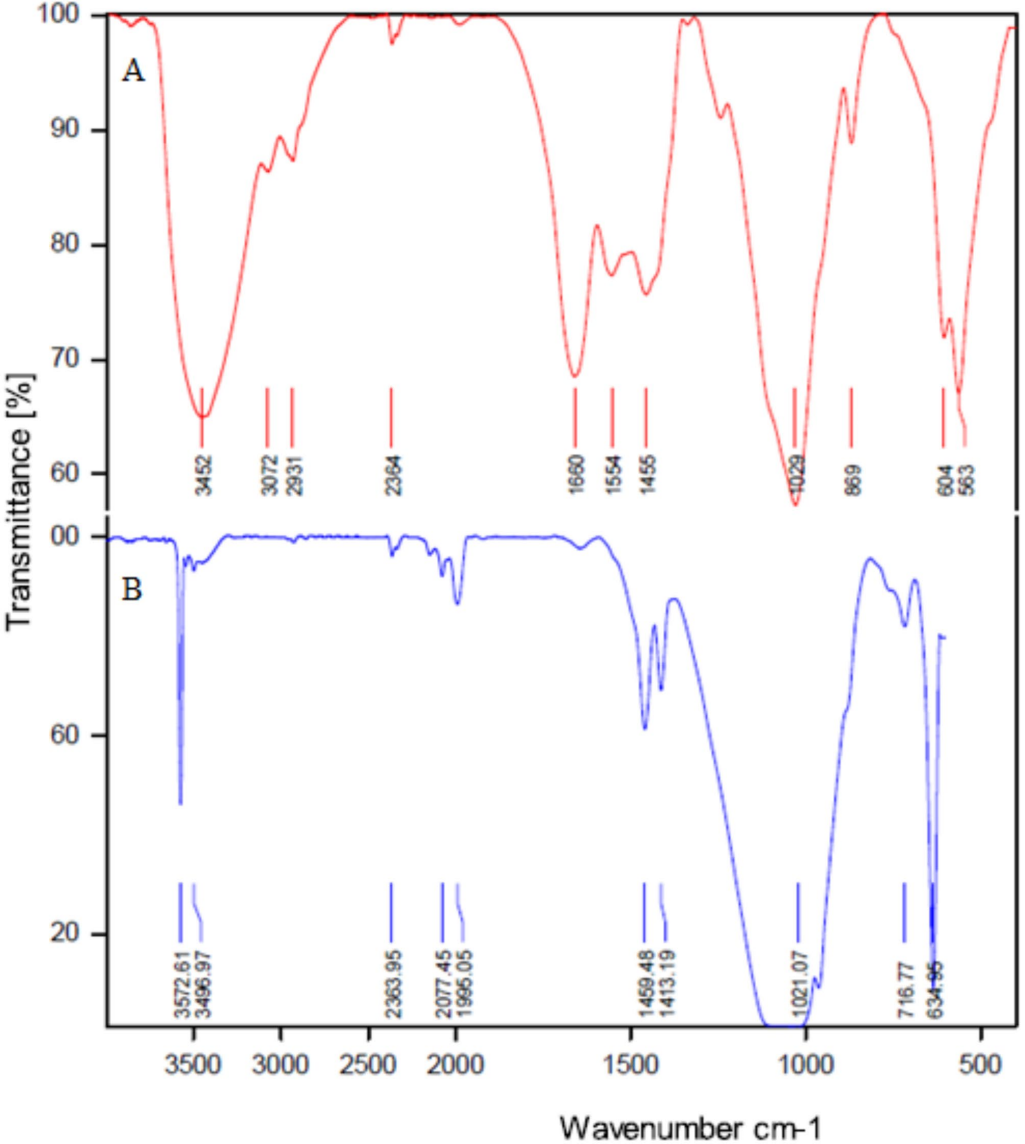
FTIR –Sheep teeth ash (HA-TSh) A: 900 ℃. B: 1150 ℃.

In goats, sheep, and cow ash, there are a series of absorption bands in the mid-region of infrared waves. These include a strong stretching band at a wavenumber of 1097 –1029 cm^− 1^ and a distinct bending absorption band at a wavenumber of 639 –427 cm^− 1^, which is associated with PO_4_^3−^. A series of minor changes is observed with increasing temperature. The absorption band at the wavenumber of 1632–3061 cm^− 1^ diminishes with increasing temperature, which can be attributed to the elimination of carbon in dental ash in the form of carbon dioxide at temperatures of 900 and 1150 ℃. This phenomenon can be observed by changing the color of teeth ash from black to white. The vibrational absorption band peaks at 565–716 cm^− 1^ and a broad absorption band at wave numbers 3439–3570 cm^− 1^, which is associated with the elongation of hydroxyl ions, becomes more stable and broader. This phenomenon is shown by the dihydroxylation of hydroxyapatite at temperatures above 900 and 1150 °C in Figs. 4, 5 and 6. The results of the infrared analysis in this study revealed that carbonated hydroxyapatite was synthesized from teeth ash at a temperature of 900 °C. The optimal temperature for converting teeth ash to carbonate-free hydroxyapatite is 900 °C. Consequently, the hydroxyapatite obtained at this temperature is referred to as natural hydroxyapatite (NHA)^49^.

In the FTIR spectroscopy of HA extracted from goat teeth and doped with Cu^2+^ and Ag^+^, new peak levels or changes in the existing peak levels can be observed. In the HA sample doped with Cu^2+^ - Ag^+^, the secondary phase was identified to contain β-calcium triphosphate (β-TCP), which could be attributed to the degradation of HA caused by chemical impurities. Infrared spectroscopy of the HA-doped Cu^2+^ - Ag^+^ sample revealed bands ranging from 900 to 1130 cm^− 1^ (960.55, 1056.99, 1093.64 cm^− 1^), indicating the stretching of the characteristic vibrational mode for the PO_4_^3−^ group of apatite β-TCP. While those from 500 to 650 cm^− 1^ (569, 601.79, and 632.65 cm^− 1^) are attributed to the bending band of the PO_4_^3−^ group^50^.

**Fig. 7:**
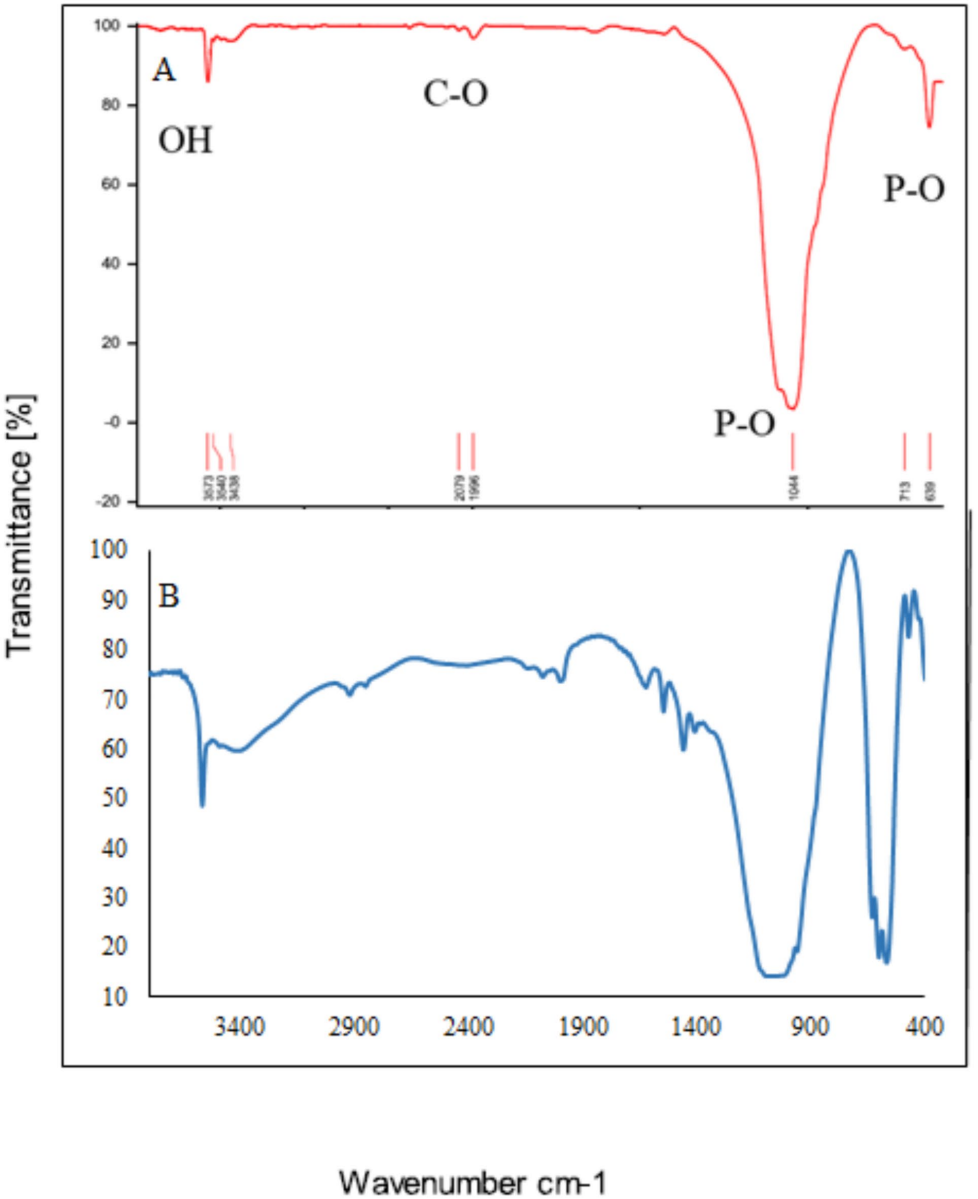
FTIR – A: Goat teeth ash (HA Pure) at 1150 ℃ B: HA doped with Cu^2+^ and Ag^+^ (HA doped).

The groups in β-TCP are detectable when silver is present in the sample. The diffract grams and infrared spectroscopy spectrum results confirm that the materials obtained by the Pechini method are biphasic^51^. The bands corresponding to stretching and bending vibrations at 3570.24 and 632.65 cm^− 1^ are attributed to the structural –OH of HA. The P-O stretching and bending vibrational bands are identified in the regions of 960.55, 1056.99, and 1093.64 cm^− 1^. The bands at 472.56 and 569 cm^− 1^ are associated with the bending vibrations of O–P–O. Additionally, the low-intensity bands observed at 1409.96 and 1460.11 cm^− 1^ are assigned to the CO_3_^2−^ group. According to the FTIR results in Fig. 7, wider bands can be observed in the samples when Cu^2+^ is present in HA^29,52^. Ag_2_O, Ag_3_PO_4,_ and, CuO peaks were not observed^53^.

#### Inductively coupled plasma mass spectrometry (ICP-MSI)

Using an ICP-OES device with a PPM detection limit, the percentage of calcium and phosphorus in hydroxyapatite extracted from goat teeth was measured at 1150 °C (pure sample of hydroxyapatite), as shown in the Table 1.^1^

**Table 1:** Ca^2+^/P ratio of hydroxyapatite extracted from Goat teeth at 1150 °C in PPM.

Samples	Compound	Amount	Ca/*P*
Goat teeth at 1150 °C	Ca^2+^	314989.3	1.45
	P	217225.0	

#### X-ray diffraction (XRD) spectroscopy analysis

**Fig. 8:**
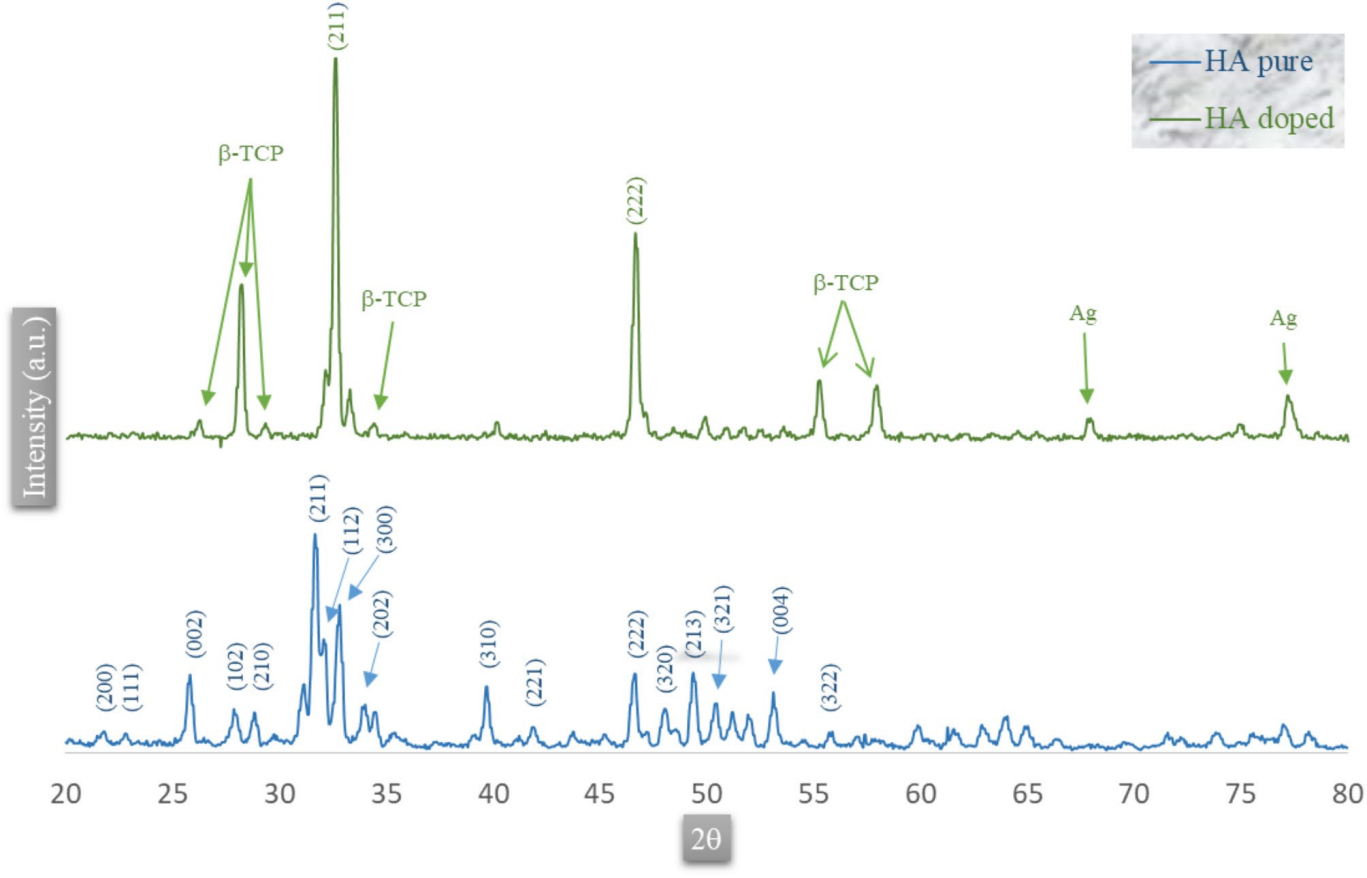
XRD, Hydroxyapatite prepared from goat teeth ash at 1150 °C and doped with Cu^2+^ and Ag^+^.

Figure 8 displays X-ray diffraction patterns of hydroxyapatite extracted from goat teeth and its doping with Cu^2+^ and Ag^+^ metals. The characteristic peaks of the hydroxyapatite (HA) for 2θ, which are found at 25.73, 28.75, 31.66, 32.09, 32.72, 33.87, and 39.65, correspond to the crystallographic planes (002), (210), (211), (112), (300), (202), and (310), respectively. Within the XRD pattern of hydroxyapatite, two primary characteristic peaks appear at 2θ angles: 25.73 and 31.66, corresponding to (002) and (211) planes, individually. Distinctive characteristic peaks with lower peak intensity at 2θ angles of 32.72, 46.57, and 53.09 are related to planes (300), (222), and (004) separately. All the diffraction peaks were observed as a phase group with a hexagonal crystal structure of p63/m, consistent with the standard card data (JCPDS No. 09-0432)^26^. The sol-gel process synthesized the materials, directly influencing the obtained phases’ composition. In the sol-gel process, copper and silver ions are uniformly distributed in the precursor liquid (sol) and lead to the formation of new crystal structures. In this process, atoms and ions are uniformly placed in the hydroxyapatite crystal structure, which leads to the creation of different phases, such as β-TCP. In the hydroxyapatite (HA) doped with Cu^2+^ - Ag^+^, a secondary phase containing beta-calcium triphosphate (β-TCP) was identified, which may be attributed to HA degradation caused by chemical impurities^54^. Biphasic calcium phosphate (BCP) is a material composed of HA and β-TCP. In samples doped with copper (Cu²⁺) and silver (Ag⁺), hydroxyapatite is identified as the primary phase. Still, the β-TCP phase is also formed as a secondary phase, especially at high temperatures. Copper and silver ions are uniformly distributed in the hydroxyapatite structure during the sol-gel process. These ions can lead to the formation of β-TCP by causing changes in the crystal structure of hydroxyapatite. As a result, these two phases (HA and β-TCP) are simultaneously present in the final structure^55^. The characteristic peaks of Ag_2_O, Ag_3_PO_4_, and CuO were not observed. This result confirms the uniform distribution of Cu^2+^ and Ag^+^ ions within the crystal structure of HA. In addition, the molar fractions of Cu^2+^ and Ag^+^ ions were tiny compared to the concentration of HA within the samples. This could explain the absence of peaks for Ag_2_O, Ag_3_PO_4_, and CuO^56,57^. Comparative outcomes have been reported in previous studies. For β-TCP, the main prominent peaks occur at 2θ angles of 26.27, 28.21, and 34.39 ℃, corresponding to the (1010), (2114), and (220) crystallographic planes, respectively, as identified in PDF file number 0169-09. Subsequently, we obtained a biphasic material, a mixture of HA and β-TCP. The prominent peaks of elemental silver were distinguished at 2θ angles of 67.86 and 77.14, corresponding to the (220) and (311) crystallographic planes as indicated in the PDF file 0783-04. This silver appears to be in its third phase, as it does not enter the crystal lattice of any unidentified apatite. Despite that, there is an alternative possibility that silver stimulates the formation of the β-TCP phase. Since the recognized peaks correspond to the HA phase, it is conceivable that Cu^2+^ has entered the HA crystal lattice. This is often because it is not observed in other peaks of the XRD pattern in Fig. 8^53^. At 1150 °C, hydroxyapatite transforms into the β-TCP phase, which is observed in XRD patterns. Hydroxyapatite is usually stable at lower temperatures, but as the temperature increases, thermal processes can lead to the formation of β-TCP. At 1150 °C, conditions are created to form β-TCP from hydroxyapatite. This phase change process has been observed in many similar calcium phosphate materials and is known as a natural phase change. This study uniformly introduced copper (Cu²⁺) and silver (Ag⁺) metal ions into the hydroxyapatite crystal structure. When these ions are incorporated into the hydroxyapatite crystal lattice, they can affect the spacing between crystal planes, and these changes cause shifts in the positions of diffraction peaks. These shifts are usually observed as slight changes in the 2θ angle in the XRD patterns, indicating that the metal ions have been introduced into the hydroxyapatite crystal structure. In particular, in this study, in the copper- and silver-doped samples, the prominent diffraction peaks of hydroxyapatite, which generally appear at specific positions in the XRD pattern, may undergo slight shifts due to the presence of these ions. These are the prominent hydroxyapatite (HA) peaks naturally shifted by metal ions. Therefore, the significant diffraction peaks observed in the pattern are probably the same hydroxyapatite peaks with slight changes in the peak positions due to the presence of copper and silver metal ions in its crystal lattice. This can be proposed to explain the observed peaks of new phases. However, considering the uniform distribution of copper and silver ions in the hydroxyapatite structure, the changes in the XRD peaks could be due to structural shifts due to the presence of these ions. These changes are partially visible, especially when there is a low ion concentration, but the hydroxyapatite structure is still largely preserved. Finally, the presence of metallic silver (Ag) detected in the XRD patterns is related to metallic silver at 2θ angles of 67.86 and 77.14 degrees, which are specifically related to the (220) and (311) planes of metallic silver. These peaks indicate the presence of metallic silver as a distinct phase alongside other phases, such as HA and β-TCP, referenced in the source (PDF No. 04-0783). These peak identifications were closely compared with known standards, and it was determined that silver appears metallic in this phase of the material’s structure (58).

#### TGA chart analysis

**Fig. 9:**
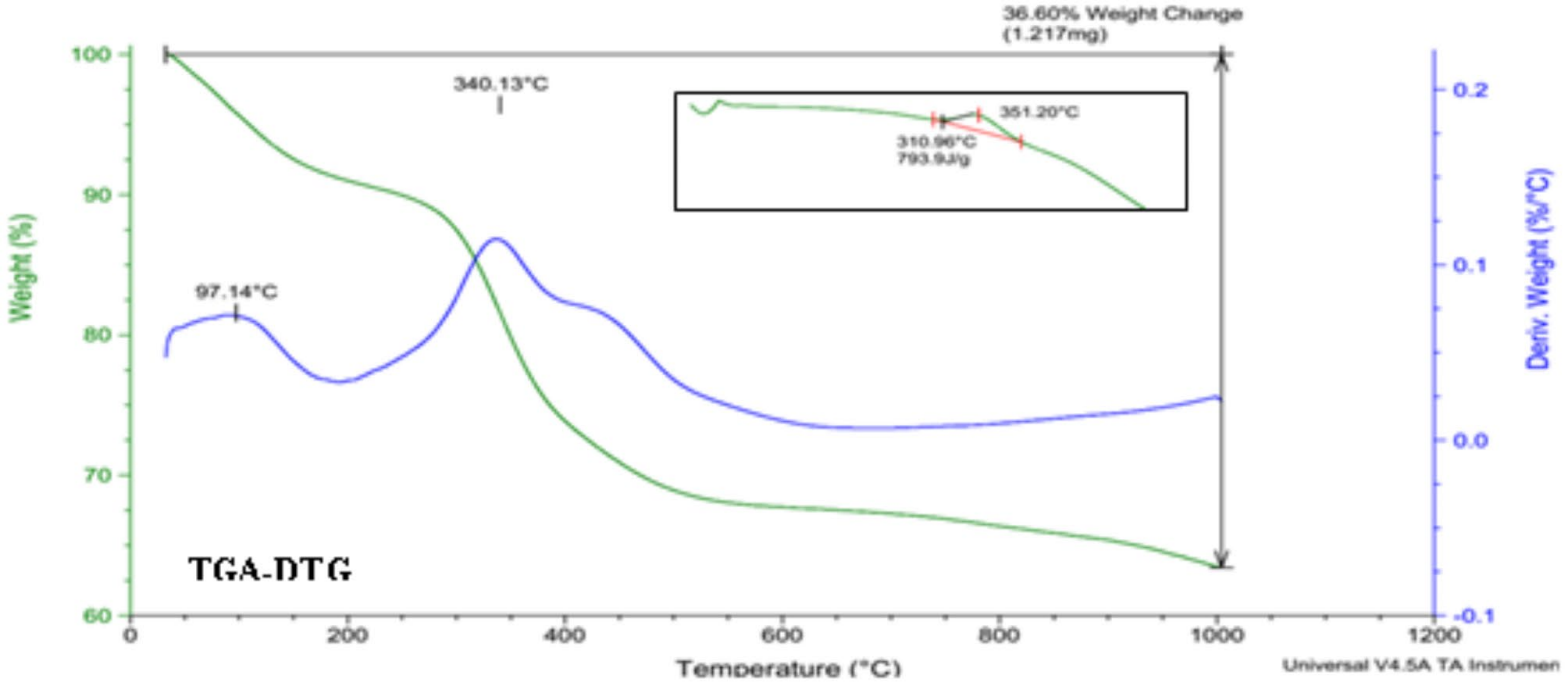
TGA-DTG analysis of Goat teeth at 1150 ℃. The insert is a DSC-TGA analysis of goat teeth at 1150 ℃.

Mass changes in goat teeth were analyzed using TGA-DTG and DSC diagrams. In the TGA-DTG curves, two inflection points were observed at 97.14 °C and 340.13 °C, corresponding to the removal of water and organic matter, respectively. In DSC, the graph indicated that the turning point for removing organic material from the teeth occurred at 351.20 °C. At 700 °C, organic materials such as collagen, lipids, keratin sulfate, and chondroitin sulfate were removed. At 1000 °C, a slight weight loss was observed, which may be due to nitrogenous and sulfurous compounds in the tooth that are released during the thermal decomposition process. Shown in Fig. 9^2^.

### Antibacterial test results of hydroxyapatite doped with Cu2+ and Ag+ nitrate

**Fig. 10:**
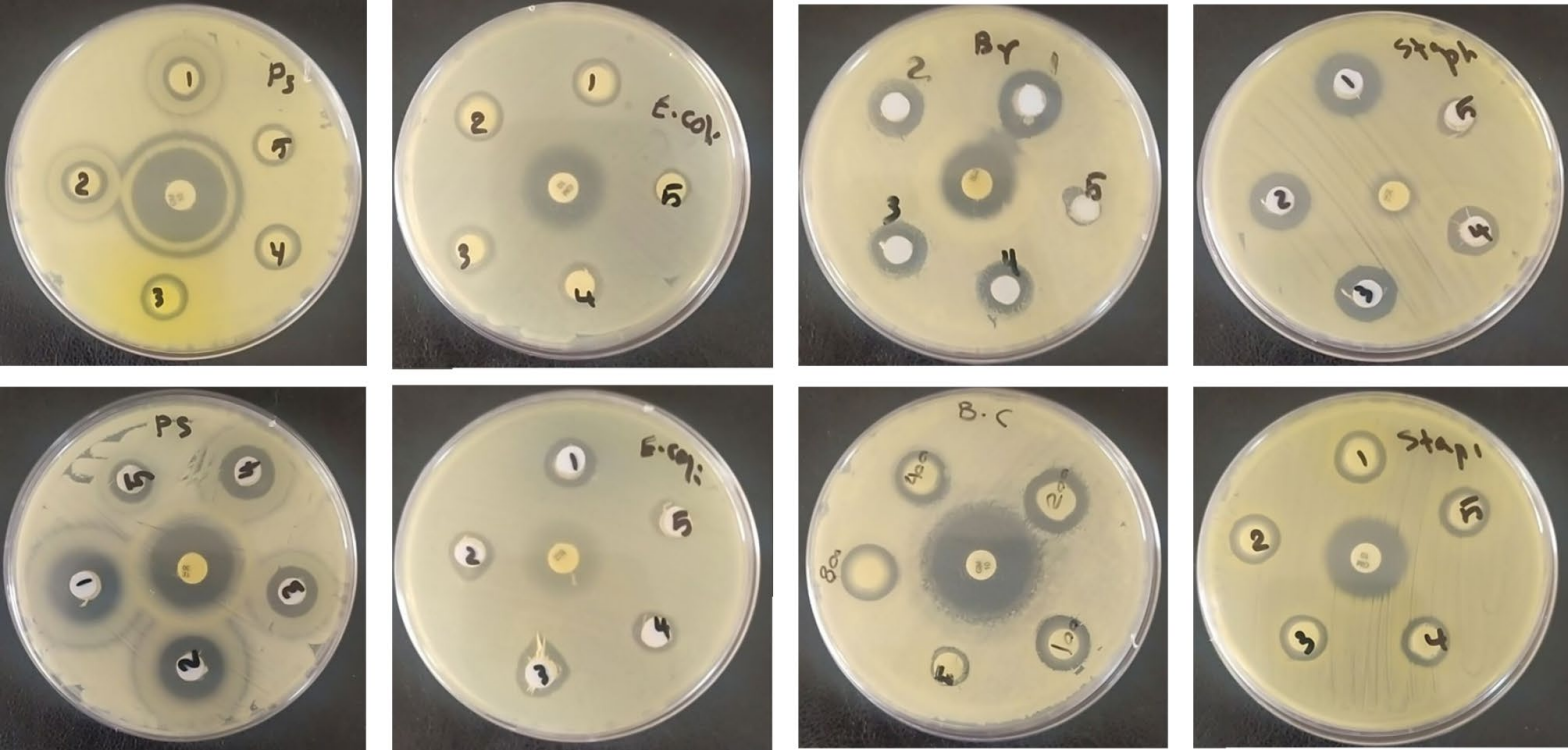
The result of lack of growth hale of gram-positive and negative bacteria against hydroxyapatite extracted from goat teeth and doped with Cu^2+^ and Ag^+^.

The results of this study showed that the antimicrobial effect of the substance depends on the concentration. With the increase in the concentration of hydroxyapatite extracted from goat teeth doped with Cu^2+^ and Ag^+^, the diameter of the halo of bacterial growth also increased Fig. 10^48^.

### The results of determining the minimum inhibitory concentration (MIC) and minimum bactericidal concentration (MBC) for hydroxyapatite doped with Cu2+ and Ag+

The antimicrobial activity and ion release properties of hydroxyapatite extracted from goat teeth doped with Cu²⁺ and Ag⁺ were systematically evaluated.

#### Quantitative antimicrobial activity (MIC and MBC)

This study’s inhibitory effect of hydroxyapatite extracted from goat teeth doped with Cu²⁺ and Ag⁺ on bacterial growth was quantitatively assessed by measuring the inhibition zone diameter at different concentrations. The results are summarized in Tables 2 and 3. The data indicate that as the concentration of hydroxyapatite doped with Cu²⁺ and Ag⁺ increases, the diameter of the inhibition zone significantly expands, demonstrating a dose-dependent antibacterial effect. At the highest concentration tested, the material exhibited the strongest inhibitory effect against Pseudomonas aeruginosa and Staphylococcus aureus. These findings further confirm the potent antimicrobial properties of hydroxyapatite doped with Cu²⁺ and Ag⁺ and align with the results obtained from MIC and MBC assays.

As the concentration of the doped hydroxyapatite increased, the inhibition zone diameters expanded significantly, indicating a potent antibacterial effect. The material’s antimicrobial activity was comparable to gentamicin for Gram-positive bacteria at higher concentrations.

**Table 2:** The results of minimum inhibitory concentration (MIC) and minimum lethal concentration (MBC) for hydroxyapatite extracted from goat teeth and doped with Cu^2+^and Ag^+^.

The name of the microorganism	Minimum inhibitory concentration (mg/ml)	Minimum lethal concentration (mg/ml)
Escherichia coli	200	800
Pseudomonas aeruginosa	50	400
Bacillus cereus	100	800
Staphylococcus aureus	50	400

**Table 3:** Diameter of the zone of inhibition at different concentrations of the bacteria studied.

Bacteria	Concentration [mg/mL]					
	10^− 1^	10^− 2^	10^− 3^	10^− 4^	10^− 5^	10^− 6^
Escherichia coli	12	10	8	7	5	3
Staphylococcus aureus	11	9	8	6	4	0
Bacillus cereus	12	10	10	8	7	6
Pseudomonas aeruginosa	15	13	10	8	7	4

#### Controlled Ion Release under physiological conditions

To evaluate the potential for sustained antimicrobial activity, the release of Cu²⁺ and Ag⁺ ions from the doped hydroxyapatite was measured after 24 h of incubation at physiological pH (7.4). Using atomic absorption spectroscopy, the concentrations of released ions were determined as follows Table 4:

**Table 4:** Concentration of ions released from doped hydroxyapatite.

Metal Ion	pH	Time [house]	Concentration Released [ppm]
Silver [Ag^+^]	7.4	24	1.386
Copper [Cu^2+^]	7.4	24	0.1186

The results indicate a controlled release of antimicrobial ions, with silver ions being released in higher concentrations than copper ions. This behavior supports the hypothesis that the doped hydroxyapatite can provide sustained antimicrobial effects, making it suitable for biomedical applications such as coatings for implants or wound dressings.

The strong antimicrobial activity and controlled ion release underscores the doped hydroxyapatite’s dual function. The MIC and MBC results confirm its immediate antibacterial efficacy, while the ion release profile suggests a prolonged effect under physiological conditions. These findings demonstrate the material’s potential to address acute and long-term microbial challenges in medical applications^48,49^.

## Results and discussion

HA is a crucial mineral in the human body, playing a significant role in the structure and function of bones and teeth, as well as in various medical and dental applications. The thermal decomposition method is an accessible, non-toxic, and low-cost technique. In addition to increased costs and time, the process of producing hydroxyapatite by chemical methods is characterized by environmental pollution and potential toxicological effects^4^.

Hydroxyapatite obtained by the thermal decomposition method is white. It is better than synthetic hydroxyapatite and has high transparency, which can be considered one of the advantages of this method^4^.

One of the advantages of thermal decomposition is that in the case of infectious diseases in the bones or teeth of animals, the high decomposition temperature destroys this contamination and disease. Moreover, the higher the temperature, the larger the particle size^59^.

HA crystals can generally exist in various forms, such as needles, rods, spheres, etc., and are usually synthesized from natural bone ash or synthetic chemicals. The properties, efficiency, phase purity, and size distribution of HA extracted from natural sources, especially from bone, depend on the extraction technique, calcination temperature, and the nature of the bones^60^.

Extracted hydroxyapatite from goat and sheep teeth at a temperature of 900–1150 °C in a shorter time (2 h) than other methods, which typically require a longer duration for preparation.

Hydroxyapatite alone does not have antibacterial properties and must be doped with metals to acquire antibacterial properties^61^.

Cu^2+^ and Ag^+^-doped hydroxyapatite (HA) is a composite material that has attracted significant attention in the field of biomaterials. Cu^2+^ and Ag^+^ ions are incorporated into HA, demonstrating several advantages such as enhanced antimicrobial properties and osteogenic activities. Composite materials exhibit antimicrobial activity as one of the main advantages of Cu^2+^ and Ag^+^ doping^48^.

Cu^2+^ and Ag^+^ ions exhibit potent antimicrobial properties, effectively inhibiting the growth of various bacteria, fungi, and certain viruses when incorporated into HA compounds. Therefore, Cu^2+^ and Ag-doped hydroxyapatite is especially well-suited for orthopedic implants and dental materials, playing a crucial role in infection prevention. Cu^2+^ and Ag^+^ ions have been shown to promote bone formation and regeneration. Cu^2+^ is an important mediator of angiogenesis, collagen production, and bone differentiation. Additionally, it improves bone mineralization and promotes the proliferation of bone cells^62,63^.

Several methods were used for the manufacture of Cu^2+^ and Ag^+^ doped hydroxyapatite, including combined precipitation and sol-gel techniques, as well as electrochemical deposition technology. These methods allow for accurate control of the concentration and distribution of doping with Cu^2+^ and Ag^+^ ions in the hydroxyapatite matrix. The choice of manufacturing methods depends on the requirements of the material characteristics, the application requirements, and the scalability^58,63^.

Doping induces the formation of crystals by Cu^2+^ and Ag^+^ ions. In doping, Cu^2+^ and Ag^+^ ions were introduced into the hydroxyapatite network to facilitate crystal formation. Doped hydroxyapatite with Cu^2+^ and Ag^+^ has antimicrobial properties, releasing Cu^2+^ and Ag^+^ ions with intrinsic antimicrobial activity. These substances are useful in antimicrobial coatings, bone transplants, and materials to prevent colonization and infections of bacteria^48,64^.

Doped hydroxyapatite is characterized by its composition, crystallization, morphology, and distribution of impurities. The graphite crystallization network indicates that copper penetration takes place within the hydroxyapatite crystallization network^65,66^.

The effect of hydroxyapatite doped with Cu^2+^ and Ag^+^ in a dose-dependent manner causes a significant increase in the halo diameter and prevents the growth of gram-positive bacteria compared to the gram-negative control group^67^.

Bacterial resistance to antibiotics is a prevalent issue in medicine. This study investigated the antibacterial effect of Cu^2+^ and Ag^+^-doped hydroxyapatite on four pathogenic bacteria: Escherichia coli, Pseudomonas aeruginosa, Staphylococcus aureus, and Bacillus aureus^33,68^.

## Conclusion

Hydroxyapatite powder, which is widely used in the fields of orthopedics and dentistry, was extracted from the teeth of domestic animals such as goats, sheep and cows by thermal decomposition method at a temperature of (1150 ℃). Then hydroxyapatite extracted from goat teeth was doped with copper and silver metals to increase its antibacterial properties and be used as biological materials for various biomedical applications. Copper and silver ions are incorporated into the hydroxyapatite network to increase the antimicrobial properties and osteogenic activity of the composite materials. These properties are particularly useful in orthopedics and dentistry, where infection prevention and bone regeneration are key. Hydroxyapatite doped with copper and silver are produced using different techniques to control the concentration and distribution of doping. These doping represent significant advances in biological materials and offer a wide range of effective solutions to issues related to infection control and bone regeneration. Further research and development in this field will open new opportunities for the use of composite materials in clinical applications, which will ultimately benefit patients and improve their quality of life. Antimicrobial results showed that hydroxyapatite doped with copper and silver prevents the growth of bacteria. Compared to other substances, they show specific antimicrobial properties against Pseudomonas and Staphylococcus aureus species.

## Data Availability

Data availability statementThe datasets used and/or analyzed during the current study are available from the corresponding author upon reasonable request.

## Declarations

### Arrive guidelines

As mentioned previously, since the study did not involve any live animal experiments, it falls outside the scope of the ARRIVE guidelines (Animal Research: Reporting of in Vivo Experiments). As such, ethical approval from an institutional or licensing committee was not required, and all methods adhered to relevant national regulations regarding the use of animal byproducts in research.
